# Modulation of growth performance, gut morphometry, and cecal microbiota in broilers by clove (*Syzygium aromaticum*) and tulsi (*Ocimum sanctum*) supplementation

**DOI:** 10.1016/j.psj.2022.102266

**Published:** 2022-10-20

**Authors:** Rafiqul Islam, Nasrin Sultana, Sonali Bhakta, Ziaul Haque, Alamgir Hasan, Mahbubul Pratik Siddique, Mohammad Rafiqul Islam

**Affiliations:** ⁎ Department of Anatomy and Histology, Bangladesh Agricultural University, Mymensingh 2202, Bangladesh; † Department of Microbiology and Hygiene, Bangladesh Agricultural University, Mymensingh 2202, Bangladesh

**Keywords:** broiler, clove, tulsi, growth performance, gut health

## Abstract

In an epoch of the growing risk of antibiotic resistance, there is a dire need to establish an effective novel feeding practice for broiler nutrition as an alternative to antibiotics. Hence, the aim of the current study was to evaluate the impact of clove powder and tulsi extract on the growth performance, gut morphologic and morphometric indices, and cecal microbial status of broiler, as an alternative to antibiotic growth promoters (**AGP**s). Sixty day-old chicks of Cobb-500 strain were randomly divided into 4 groups, each having 15 birds. Chicks of the control group (T0) were fed commercial broiler feed with no additional supplementation. The treatment groups were offered commercial broiler feed and received clove powder and tulsi extract with drinking water at the rate of 0.5% + 2% (T1), 1% + 3% (T2), and 1.5% + 4% (T3), respectively. Results showed a nonlinear relationship with the dosage of clove and tulsi. All the growth parameters substantially (*P* < 0.05) improved in T2 while T1 and T3 showed no significant improvement compared to T0. The final body weight was significantly (*P* < 0.05) higher in T2. Giblet and offal weights showed no noticeable differences except in the intestine and heart where intestine weight markedly (*P* < 0.05) decreased in T3 and heart weight significantly (*P* < 0.05) increased in T1 and T2. Clove and tulsi supplementation substantially improved the villus height and villus surface area of the small intestine in T2 while the large intestine remained mostly unaffected by the treatment. Cecal microbial status significantly improved in all the treatment groups having increased (*P* < 0.05) *Lactobacillus* spp. count and decreased (*P* < 0.05) *E. coli* count compared to T0. Based on the aforementioned findings, it can be concluded that the combination of clove and tulsi can improve the growth performance and gut health of broilers which is largely dose-dependent and might be supplied as a potential alternative to AGPs.

## INTRODUCTION

It is anticipated that there will be an approximately 44 Mt increase in the world's meat production, reaching 373 Mt total meat production by 2030; 84% of which will take place in developing countries (OECD/FAO, 2021). Even though it might expand more slowly than it did over the past decade, poultry meat will still be the major player in the growth of meat production. Worldwide poultry meat consumption is expected to reach 152 Mt by 2030, making up 52% of the total increase in meat consumption (OECD/FAO, 2021). Poultry farmers can respond promptly to market signals because of astonishing feed efficiency and shorter production period. Consumers also prefer poultry meat due to its lower price and higher protein-to-fat content. Hence, the productive performance of poultry species is one of the major interests of livestock production scientists.

There are plenty of feed additives available in the market, all of which claim to improve growth performance (Dhama et al., 2014). In the past few decades, poultry industries have greatly benefited from the use of antibiotics in diets as growth promoters in terms of feed efficiency and growth rate. In order to maintain a healthy gut and manage subclinical infections, antibiotics have been used as feed additives in the poultry industry (David et al., 2012). But since 2006, certain regions, including the European Union, have prohibited the use of antibiotics as growth promoters due to the rising concerns about antibiotic resistance and its residual effects on meat (Wierup, 2001). However, this solution will not be feasible without introducing an effective alternative to antibiotic growth promoters (**AGP**s). Therefore, researchers are evaluating biological products like enzymes, organic acids, amino acids, probiotics, and prebiotics as potential alternatives to AGPs in the poultry diet (Agostini et al., 2012; Laudadio et al., 2012; Dhama et al., 2014; Chowdhury et al., 2018; Thuekeaw et al., 2022). Previous scientific evidence had already validated that by providing optimum conditions, without using AGPs, the desired growth rate can be attained (Engster et al., 2002).

Clove, tulsi, ginger, turmeric, aloe vera, garlic, neem, oregano, anise, cinnamon, and amla are popularly known plants or plant products used in the diet to improve feed efficiency and growth performance of poultry (Kreydiyyeh et al., 2000; Ertas et al., 2005; Eevuri and Putturu, 2013; Cabuk et al., 2014; Mohammadi et al., 2014; Mustafa, 2016; Jahejo et al., 2019; Arif et al., 2022). These plants contain various bioactive ingredients like glycosides, alkaloids, flavonoids, mucilage, and bitters (Baliga et al., 2013; Eevuri and Putturu, 2013; Batiha et al., 2020). Nowadays, herbal plant-derived phytobiotics are gaining more attention for their potential antimicrobial role as alternatives to AGPs. These phytobiotics can play a game-changing role in organic livestock production. Herbal medicines (like clove and tulsi) can be an affordable feed additive for any farming level, especially for marginal farmers (El-Shall et al., 2022). Clove is one of the popular phytochemical-rich plants that reportedly improves the gut health and growth performance of poultry and can be used alone or in combination with other plant extracts (Batiha et al., 2020; Arif et al., 2022). It has potential antimicrobial, antioxidant, anti-inflammatory, anesthetic, and antidiabetic effects (Milind and Deepa, 2011; Batiha et al., 2020). On the other hand, tulsi (*Ocimum sanctum*) is rich in essential oils (i.e., eugenol and linalool) and organic acids (i.e., prunol, b-caryophyllene, and labiatenic acid) (Eevuri and Putturu, 2013). These bio-active components of tulsi have made it the mother medicine of nature having antimicrobial, anti-stress, anti-inflammatory, immunomodulatory, antipyretic, antiasthmatic, hypoglycaemic, hypotensive, and analgesic effects (Baliga et al., 2013; Eevuri and Putturu, 2013). Clove and tulsi also play roles in the improvement of the gastrointestinal ecosystem mostly through inhibition of pathogenic microorganisms' growth and modulating the gut morphologic and morphometric attributes (Agostini et al., 2012; Wati et al., 2015; Chowdhury et al., 2018; Chakma et al., 2020). There are abundant resources on the individual effect of tulsi and clove in poultry species. However, there is no report on the combined effects of clove and tulsi on the growth performance and gut health of broilers.

In light of the aforementioned remarks, we hypothesized that combined supplementation of clove and tulsi to broiler drinking water would alter their growth performance, morphologic, and morphometric indices of the gut as well as the microbial community of the cecum. The current study was designed to investigate the combined effects of clove powder and tulsi extract on the aforecited attributes.

## MATERIALS AND METHODS

### Statement of the Experiment

The experimental trial and laboratory works were carried on at the Department of Anatomy and Histology, Bangladesh Agricultural University (**BAU**), Mymensingh. Animal Welfare and Experimentation Ethics Committee (AWEEC), BAU gave ethical clearance to conduct the experimental trial [AWEEC/BAU/2021(05)].

### Housing and Management of Experimental Broilers

Following a thorough clean-water wash, the experiment shed and broiler cages were disinfected with 3% potassium permanganate spray. Broiler feeding trays and drinkers were also washed with 1% bleached water followed by a thorough clean water washing and sun-drying. All the utensils were then placed inside the experimental shed for further disinfection by fumigation (17.5 g of potassium permanganate and 35 ml of 37% formaldehyde) and the shed was sealed for 48 h to ensure complete disinfection. Finally, the shed was reopened to remove any toxic gases.

Nourish Poultry & Hatchery Ltd., Mymensingh, Bangladesh provided us with 60 unsexed day-old Cobb-500 broiler chicks for the current study. After seven days of acclimatization period, the chicks were allocated into four experimental groups at random (15 birds/group). Then each experimental group was kept in individual iron cages (Size: 5 × 4 × 2.5 feet) and maintained under identical conditions. Standard brooding (35°C for the first 3 d followed by a gradual decrease of about 3°C daily until reached 21°C) and rearing temperature (21°C), and relative humidity (50–60%) were maintained throughout the experiment (Islam et al., 2021).

### Bio-security Measures

This experiment was conducted under strict bio-security measures. Visitors were restricted from entering the experimental shed. Cleaning of the feeders and drinkers with 1% bleached water was done on a daily basis to prevent microbial growth. The broilers were also vaccinated against Newcastle Disease (BCRDV vaccine, Bangladesh Livestock Research Institute, Bangladesh) at 5 d of age and Infectious Bursal Disease (GUMBORO D78, Intervet, India) at 11 d of age.

### Preparation of Clove Bud Powder and Tulsi Extract

The clove (*Syzygium aromaticum*) buds were properly sun-dried and ground into very fine powder. Then the powders were sieved using a 25-unit mesh diameter sieve. Finally, the clove powder was stored in an airtight container for later use. Aqueous extract of tulsi (*Ocimum sanctum*) was prepared on a daily basis using fresh leaves. The leaves were properly blended in water and then an adequate amount of water was added to prepare a 2%, 3%, and 4% aqueous extract of tulsi.

### Experimental Diets

All the experimental groups were offered a balanced diet purchased from Nourish Feeds Limited, Mymensingh, Bangladesh, and fresh drinking water (Islam et al., 2022). The clove powder and tulsi extract was supplied with drinking water (from d 8 to d 28) in 3 different combinations on the basis of their concentrations.

Treatment (T) T0: Control group, did not receive any plant extract; T1: 0.5% clove and 2% tulsi; T2: 1% clove and 3% tulsi; T3: 1.5% clove and 4% tulsi. The required amount of clove powder and tulsi extract was added to the drinking water and then blended properly to prepare homogenous mixtures. The experiment was run for 28 d where the plant powders were supplied for 21 d (from d 8 to d 28).

### Growth Performance

The initial body weight of each broiler was measured on d 7 before the start of the treatment. Feed consumption was documented for each experimental group on a daily basis (from d 8 to d 28). The leftover feed, if any, was subtracted from the feed offered. The average feed intake by each bird during the treatment period was then calculated. The final body weight of each broiler was measured on d 28. Body weight gain was measured by subtracting the d 8 weight from the d 28 weight. The feed conversion ratio was calculated by the following formula:

Feed conversion ratio = $\frac{\left\{Average\;feed\;intake\;(from\;d\;8\;to\;d\;28)\right\}}{\left\{Average\;weight\;gain\;(from\;d\;8\;to\;d\;28)\right\}}$

### Sample Collection and Processing

Five broilers were ethically sacrificed (cervical dislocation technique) from each experimental group on d 14 and d 28. After immediate dissection of the sacrificed broilers, carcass weight, giblet weight, abdominal fat weight, length, and weight of different gut segments were measured. Segments (1–1.5 cm in length) of the small intestine (duodenum, jejunum, and ileum) and large intestine (cecum and colorectum) were collected and flushed with phosphate-buffered saline (**PBS**) to wash out the digesta. Collection site for each segment: Duodenum–from the midpoint of the ascending loop; Jejunum–from the mid-region between the entrance of the bile duct and Meckel's diverticulum (**MD**); Ileum–from the midpoint between the MD and ileocecal junction; Cecum–from the blind end of the right cecum; Colorectum–from the midpoint between the ceco-colic junction and rectal opening.

Intestinal segments were fixed in neutral-buffered formalin (10%) for histological study. Tissue samples were then dehydrated using rising grades of ethanol (Merk, Damstadt, Germany), cleared using xylene (Merck, Damstadt, Germany), and paraffin-embedded. Five micrometer thin tissue sections were cut from the paraffin-embedded gut tissues and finally, Hematoxylin and Eosin staining (Merck, Damstadt, Germany) was performed for histomorphometric investigation.

### Measurement of Gut Histomorphometric Indices

The morphometric indices investigated were height, width, and surface area of the villi, depth of the crypts, villi height: crypt depth (for the small intestine), and thickness of the mucosa (for the large intestine). Specific sites for the measurements: Villus height–from the crypt to the apex of the villus; villus width–at first, the width of the villus at one-third and two-thirds of its height was measured and then the average width of each villus was calculated from these two measures; crypt depth–the distance between the base of the villus to the tunica submucosa (Zang et al., 2009). The following formula was used to calculate the villus surface area: $\frac{1}{2}\left(average\;villus\;width\;\times villus\;height\right)$ (Laudadio et al., 2012). Morphometric measurements were performed on 30 randomly selected intact villi and crypts from each small intestinal segment. Mucosal thickness was measured from 10 randomly selected points of each tissue section.

### Analysis of Microbial Status in the Cecum

The cecal contents were aseptically collected from the broilers after sacrificing on d 28 of the experiment and stored at 4°C temperature in sterile containers. On the same day, the cecal contents were processed for bacteriological analysis. The cecal content (1 gm) was diluted following the 10-fold serial dilution method using sterile PBS and finally, 10^−5^ dilution was used for drop plating in the agar media. Plate Count Agar (**PCA**), Eosin Methylene Blue (**EMB**) agar, and De Man, Rogosa, and Sharpe (**MRS**) agar (purchased from HiMedia, Mumbai, India) media were used for the total bacterial count, total probiotic count, particularly *Lactobacillus* spp., and *E. coli* count, respectively. The incubation period was 24 h (37°C) except for *Lactobacillus* spp. (48 h). The colonies for each bacterial population were counted manually. The bacterial counts were presented as Log_10_ colony forming units (**CFU**) per gm of the cecal content.

### Statistical Analysis

All the datasets obtained in the current experiment were statistically analyzed (IBM SPSS Statistics, version 22) by using the one-way ANOVA technique following a completely randomized design. The unit of the analysis was the individual bird (there was no pen replication). Duncan's Multiple Range Test (**DMRT**) was done to make comparisons between the mean values. Difference was described as significant when *P* < 0.05.

## RESULTS

### Growth Performance

The combined impacts of clove and tulsi supplementation on the broiler growth performance are summarized in Table 1. The initial weights of the broilers of different experimental groups were nearly identical (*P* > 0.05). Clove and tulsi inclusion markedly (*P* < 0.05) affected the feed consumption and other growth parameters of the broilers. The T2 group had substantially (*P* < 0.05) higher feed intake in comparison to the rest of the experimental groups. In line with feed intake, the T2 group had considerably higher (*P* < 0.05) final weight and average weight gain in comparison to the other groups. Both of these parameters were also substantially higher (*P* < 0.05) in the T1 group while the T0 and T3 groups had no noticeable difference (*P* > 0.05). The best feed conversion efficiency was seen in the T2 group which was followed by T1, T3, and T0. The T2 group yielded the highest carcass weight while the T3 group yielded the lowest carcass weight.

**Table 1:** Effects of clove powder and tulsi extract supplementation on the growth performance of broiler chicken.

	Treatments^1^					
Item	T0	T1	T2	T3	SEM	*P*-value
Initial body weight (d 7), g	178.7	178.5	179.3	178.1	0.26	0.734
Feed intake (d 8–d 28), g	2397^bc^	2491^b^	2642^a^	2306^c^	71.74	0.043
Final body weight (d 28), g	1577.3^bc^	1660.4^b^	1768.5^a^	1531.3^c^	52.07	0.004
Body weight gain, g	1398.7^bc^	1481.9^b^	1589.2^a^	1353.3^c^	51.84	0.001
Feed Conversion Ratio (FCR)	1.71^a^	1.68^ab^	1.66^b^	1.7^a^	0.01	0.021
Carcass weight, g	1091.7^c^	1133.7^b^	1209.3^a^	1067.6^c^	20.82	0.001

a,b,c Within a row, values with different alphabetic superscripts differ significantly (*P* < 0.05).

1 T0: represents the control group; T1, T2, and T3 groups represent supplementation of 0.5% clove + 2% tulsi, 1% clove + 3% tulsi, and 1.5% clove and 4% tulsi, respectively***.***

### Giblet and Offal

Data presented in Table 2 presents the impact of clove and tulsi supplementation on giblets and offal of broilers. The weights of the liver, gizzard, spleen, and abdominal fat depot of the broilers were unaffected by the treatments. However, the weight of the intestine decreased substantially (*P* < 0.05) in T3 while the heart weight significantly (*P* < 0.05) increased in T1 and T2.

**Table 2:** Effects of clove powder and tulsi extract supplementation on the giblets and offal of broiler chicken.

	Treatments^1^					
Items	T0	T1	T2	T3	SEM	*P*-value
Intestine, g	61.33^a^	61.23^a^	62.44^a^	56.42^b^	3.10	0.002
Liver, g	47.28	48.78	51.27	46.45	2.26	0.233
Heart, g	6.99^b^	7.98^a^	8.32^a^	6.91^b^	0.45	0.030
Gizzard, g	18.87	20.99	22.26	19.06	1.21	0.065
Spleen, g	1.98	1.88	2.04	1.77	0.17	0.441
Abdominal fat, g	32.64	33.68	37.70	38.20	2.01	0.055

a,b,c Within a row, values with different alphabetic superscripts differ significantly (*P* < 0.05).

1 T0: represents the control group; T1, T2, and T3 groups represent supplementation of 0.5% clove + 2% tulsi, 1% clove + 3% tulsi, and 1.5% clove and 4% tulsi, respectively***.***

### Gut morphologic and Morphometric Indices

**Figure 1:**
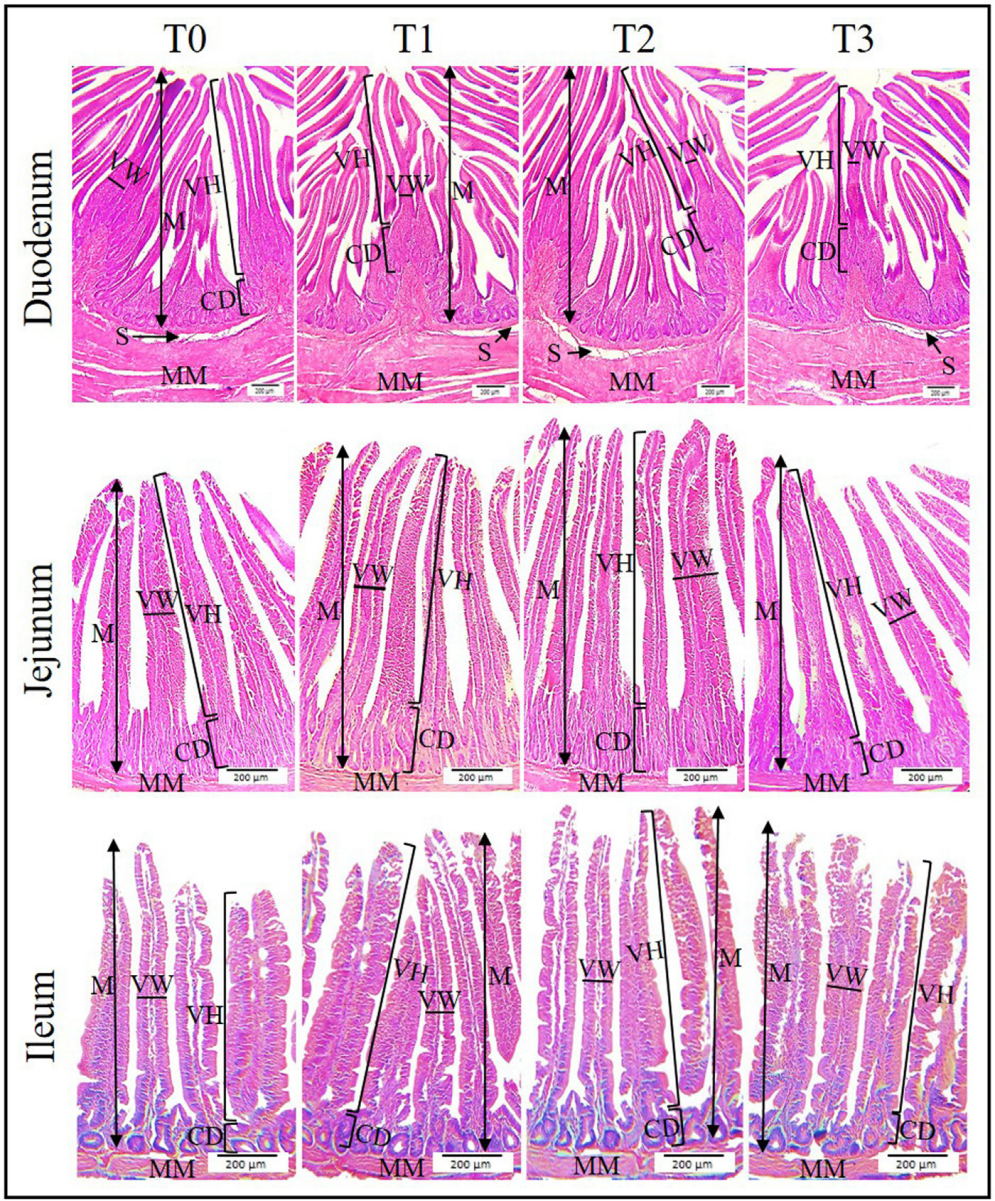
Histomorphology of the small intestine (duodenum, jejunum, and ileum) of broilers showing villus height, width, and crypt depth. The mucosa of the small intestine was thrown into finger-like projections (villi). T0: represents the control group; T1, T2, and T3 groups represent supplementation of 0.5% clove + 2% tulsi, 1% clove + 3% tulsi, and 1.5% clove and 4% tulsi, respectively**.** Magnification - 100X, Scale bar - 200 µm. CD, crypt depth; M, mucosa; MM, muscularis mucosa; S, submucosa; VH, villus height; VW, villus width.

**Figure 2:**
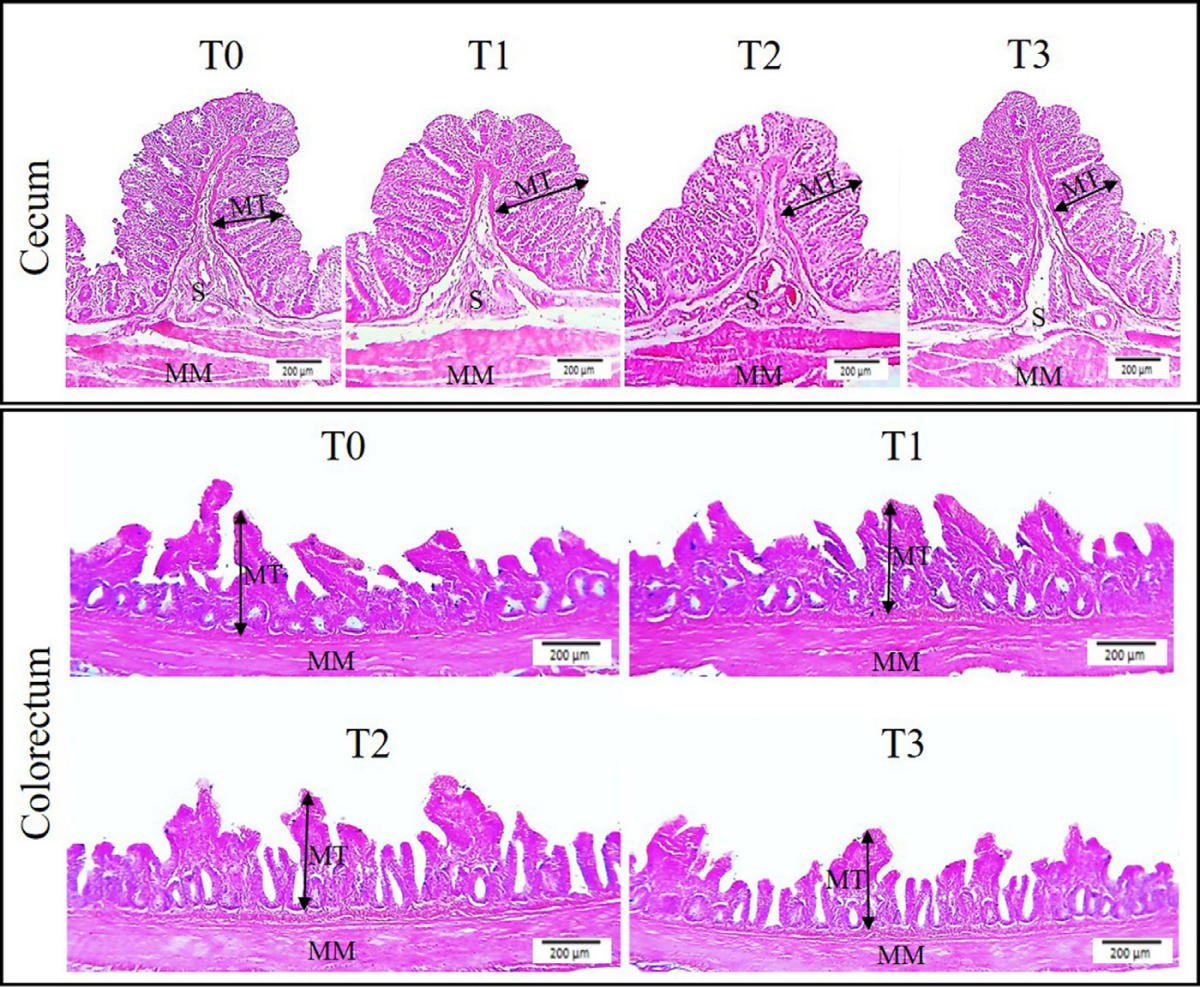
Histomorphology of the large intestine (cecum and colorectum) of broilers showing mucosal thickness. The mucosa of the small intestine was thrown into numerous folds. T0: represents the control group; T1, T2, and T3 groups represent supplementation of 0.5% clove + 2% tulsi, 1% clove + 3% tulsi, and 1.5% clove and 4% tulsi, respectively**.** Magnification - 100X, Scale bar - 200 µm. MM, muscularis mucosa; MT, mucosal thickness; S, submucosa.

The morphologic appearances of the small intestine and large intestine are presented in Figures 1 and 2, respectively. The intestine is histologically characterized by 4 distinct layers that is, mucosa, submucosa, muscularis mucosa, and serosa. The mucosa of the small intestine was thrown into villi while the mucosa of the large intestine was thrown into folds. No histomorphologic alteration was observed in the control and treatment groups.

### Small Intestine

The morphometric data of the small intestine of broilers are summarized in Tables 3 and 4. The length of the duodenum markedly (*P* < 0.05) increased in the T1, T2, and T3 in comparison to the T0 on d 14. However, on d 28, there was no substantial variation among the groups except group T3 which had considerably (*P* < 0.05) lower duodenal length compared to the other groups. The T2 group had the largest jejunal length while the T0 group had the smallest. The T1 and T3 groups had no significant (*P* > 0.05) difference. On d 14, the experimental groups had no noticeable difference (*P* > 0.05) in case of ileum length. On d 28, ileal length was almost similar in the T1 and T2 groups, though the T0 and T3 had significantly lower (*P* < 0.05) ileal lengths.

**Table 3:** Effects of clove powder and tulsi extract supplementation on small intestinal morphometry of broiler chickens on d 14.

	Treatments^1^					
Attributes	T0	T1	T2	T3	SEM	*P*-value
Duodenum						
Length, cm	18.5^b^	22.10^a^	23.40^a^	23.20^a^	1.32	0.020
Villus height, μm	937.43	948.20	980.40	941.18	26.12	0.352
Villus width, μm	168.10	171.90	182.65	166.65	10.53	0.430
Crypt depth, μm	162.85	164.68	166.08	164.70	10.38	0.992
Villus surface area, mm^2^	0.16	0.16	0.18	0.16	0.01	0.399
Villus height to crypt depth	5.77	5.80	6.06	5.79	0.29	0.711
Jejunum						
Length, cm	76.50^c^	91.33^b^	101.47^a^	92.83^b^	3.54	0.001
Villus height, μm	637.23	644.70	676.43	640.10	17.81	0.121
Villus width, μm	117.60	123.75	127.90	116.53	7.45	0.390
Crypt depth, μm	107.40	112.25	116.30	112.08	7.13	0.671
Villus surface area, mm^2^	0.08	0.08	0.09	0.08	0.01	0.453
Villus height to crypt depth	5.95	5.80	5.97	5.79	0.28	0.881
Ileum						
Length, cm	11.73	13.37	14.63	15.50	1.54	0.158
Villus height, μm	484.15^b^	502.60^ab^	539.03^a^	489.43^b^	19.64	0.036
Villus width, μm	101.28	106.55	113.20	103.43	6.56	0.305
Crypt depth, μm	97.95	102.03	108.08	98.88	6.26	0.417
Villus surface area, mm^2^	0.05	0.05	0.06	0.05	0.01	0.203
Villus height to crypt depth	4.94	4.96	5.12	5.02	0.26	0.895

a,b,c Within a row, values with different alphabetic superscripts differ significantly (*P* < 0.05).

1 T0: represents the control group; T1, T2, and T3 groups represent supplementation of 0.5% clove + 2% tulsi, 1% clove + 3% tulsi, and 1.5% clove and 4% tulsi, respectively***.***

**Table 4:** Effects of clove powder and tulsi extract supplementation on small intestinal morphometry of broiler chickens on d 28.

	Treatments^1^					
Attributes	T0	T1	T2	T3	SEM	*P*-value
**Duodenum**						
Length, cm	27.43^a^	28.27^a^	27.47^a^	24.80^b^	0.73	0.007
Villus height, μm	1261.70^b^	1274.25^b^	1311.48^a^	1253.08^b^	17.66	0.011
Villus width, μm	235.50	246.93	255.95	238.23	9.09	0.121
Crypt depth, μm	222.13	223.35	225.60	220.50	7.66	0.924
Villus surface area, mm^2^	0.30^b^	0.32^ab^	0.34^a^	0.30^b^	0.01	0.033
Villus height to crypt depth	5.69	5.72	5.83	5.74	0.17	0.862
**Jejunum**						
Length, cm	110.43^c^	126.53^ab^	130.27^a^	120.13^b^	3.82	0.004
Villus height, μm	857.90^b^	866.45^b^	891.88^a^	852.03^b^	12.02	0.011
Villus width, μm	164.93	172.93	179.15	166.88	6.34	0.123
Crypt depth, μm	151.05	151.88	153.55	149.88	5.20	0.912
Villus surface area, mm^2^	0.14^b^	0.15^ab^	0.16^a^	0.14^b^	0.01	0.043
Villus height to crypt depth	5.69	5.72	5.82	5.74	0.17	0.880
**Ileum**						
Length, cm	18.77^b^	21.77^a^	22.8^a^	18.23^b^	1.09	0.001
Villus height, μm	668.80^b^	688.20^b^	721.43^a^	676.63^b^	9.55	0.001
Villus width, μm	146.20	153.15	158.63	147.80	5.64	0.126
Crypt depth, μm	137.88	138.38	139.98	136.75	4.74	0.923
Villus surface area, mm^2^	0.10^b^	0.11^ab^	0.12^a^	0.10^b^	0.01	0.003
Villus height to crypt depth	4.86	4.99	5.17	5.00	0.15	0.244

a,b,c Within a row, values with different alphabetic superscripts differ significantly (*P* < 0.05).

1 T0: represents the control group; T1, T2, and T3 groups represent supplementation of 0.5% clove + 2% tulsi, 1% clove + 3% tulsi, and 1.5% clove and 4% tulsi, respectively***.***

Though most of the histomorphometric indices were found numerically higher in the T2 group on d 14, they were not significant (*P* > 0.05). Noticeable variation was observed on d 14 only in case of the villus height of the ileum where the T2 group had the largest villus height and the T0 and T3 groups had the lowest. Villi height was significantly (*P* < 0.05) larger in all the segments of the small intestine in the T2 group as compared to other groups on d 28. The surface area of the villus was also significantly (*P* < 0.05) greater in the T2 group in comparison to the T0 and T3 groups on d 28. No substantial dissimilarity was found between the T1 and T2 groups. The small intestinal segments had no mentionable (*P* > 0.05) difference in terms of villus width, crypt depth, and villus height to crypt depth ratio.

### Large Intestine

The morphometric data of the large intestine of different experimental groups are presented in Table 5. On d 14, the T3 group had the largest cecal length while the T0 had the smallest. Similarly, the T3 had the largest colorectal length while the T0 and T2 had the smallest. Howsoever, no mentionable (*P* > 0.05) variation was observed in cecal or colorectal length on d 28. In the histomorphometric investigation, the cecum and colorectum revealed no noticeable (*P* > 0.05) alteration in terms of mucosal thickness.

**Table 5:** Effects of clove powder and tulsi extract supplementation on large intestinal mucosal morphometry of broiler chickens.

	Treatments^1^					
Attributes	T0	T1	T2	T3	SEM	*P*-value
Cecum						
Length, cm						
d 14	9.83^c^	11.23^bc^	11.40^b^	12.90^a^	0.62	0.008
d 28	16.43	17.00	17.73	18.73	1.17	0.301
Mucosal thickness, μm						
d 14	268.88	291.05	299.83	293.78	14.53	0.178
d 28	405.33	413.43	417.33	415.25	14.51	0.852
Colorectum Length, cm						
d 14	4.63^b^	5.00^a^	4.43^b^	5.17^a^	0.12	0.001
d 28	6.67	6.7	6.57	6.43	0.24	0.706
Mucosal thickness, μm						
d 14	241.90	261.85	269.93	264.48	13.07	0.174
d 28	364.78	372.03	375.73	373.85	11.02	0.846

a,b,c Within a row, values with different alphabetic superscripts differ significantly (*P* < 0.05).

1 T0: represents the control group; T1, T2, and T3 groups represent supplementation of 0.5% clove + 2% tulsi, 1% clove + 3% tulsi, and 1.5% clove and 4% tulsi, respectively***.***

### Microbial Status in the Cecum

The microbial status in the cecum is shown in Table 6. Clove and tulsi combined supplementation did not affect the total bacterial count. Though the treatment groups had comparatively higher bacterial counts compared to the control, they were not significant (*P* > 0.05). However, the T1, T2, and T3 groups had substantially (*P* < 0.05) higher *lactobacillus* spp. population in comparison to the T0. On the contrary, the *E. coli* count was markedly (*P* < 0.05) lower in the treated groups.

**Table 6:** Cecal bacterial count (log_10_ CFU/g) of experimental broilers fed diet supplemented with different combinations of clove and tulsi.

	Treatments^1^					
Bacterial count	T0	T1	T2	T3	SEM	*P*-value
Total bacteria	6.95	7.91	7.43	7.16	0.48	0.291
*Lactobacillus* spp.	6.29^b^	8.22^a^	8.71^a^	8.47^a^	0.38	0.001
a. E. coli	6.80^a^	4.88^c^	4.38^c^	5.94^b^	0.34	0.001

a,b,c Within a row, values with different alphabetic superscripts differ significantly (*P* < 0.05).

1 T0: represents the control group; T1, T2, and T3 groups represent supplementation of 0.5% clove + 2% tulsi, 1% clove + 3% tulsi, and 1.5% clove and 4% tulsi, respectively***.***

## DISCUSSION

### Feed Intake

The results of the current study led us to the opinion that clove powder and tulsi extract supplementation, when offered in the optimum concentrations, increases broiler feed consumption. Similar findings were reported in earlier studies (Mukhtar, 2011; Agostini et al., 2012; Bhosale et al., 2015; Arif et al., 2022). Clove can be supplemented as a potential alternative to AGPs which also improves the feed intake in the broiler (Dalkilic and Güler, 2009). Vasanthakumar et al. (2013) reported increased feed intake in response to 0.5% tulsi supplementation. The improvement in feed consumption in broilers associated with the supplementation of clove and tulsi might be due to the increased appetite resulting from their antimicrobial activity, augmented secretion of enzymes that help in digestion, increased digestive performance, and intestinal absorbability (Yang et al., 2009). However, the level of feed intake is quite dose-dependent as the feed intake was found to be decreased at the higher dose of clove (1.5%) and tulsi (4%). A similar finding was also reported in some of the previous studies (Mustafa, 2016; Al-Mufarrej et al., 2019; Naeem et al., 2022). The reduced feed intake at the high dose of clove (1.5%) and tulsi (4%) might be due to the reduced palatability because of the presence of a high amount of eugenol which decreases gut motility (Daniel et al., 2009).

### Body Weight Gain

The body weight gain in the experimental broilers showed a linear relationship with feed consumption. Clove and tulsi are frequently used as growth promoters (Agostini et al., 2012; Naeem et al., 2022). Dietary supplementation of clove oil to the broilers improves their weight gain (Mustafa, 2016). Supplementation of essential oils like clove oil at the rate of 200 ppm augments the growth rate and thus results in increased body weight gain (Ertas et al., 2005). Another study reported that supplementation of 450 ppm clove essential oil substantially increases weight gain in broilers (Mehr et al., 2014). However, the efficacy of clove is dose-dependent as a negative impact on the growth performance of broilers was found in the current study in response to a higher dose (600 mg/L). Ertas et al. (2005) reported a similar effect while the dose rate was 400 mg/kg. In the current study, clove supplementation at a dose rate of 1% improved the growth performance of broilers. This finding is similar to Al-Mufarrej et al. (2019), who reported a gradual decrease in body weight gain when the dose of clove is 2% or more. Supplementation of 0.5 to 1.5% tulsi increases feed intake, body weight gain, and ultimately the growth performance of poultry (Vasanthakumar et al. 2013; Naeem et al., 2022). Clove buds comprise a high amount of saponin (Chaudhary et al., 2018). Clove supplementation reduces amino acid degradation and improves their absorption, thus contributing to increased body weight gain (Lee and Shibamoto, 2002; Mansoub, 2011). Tulsi leaf contains different active compounds like eugenol, apigeninursolic acid, rosmarinic acid, carnosol, cirsimaritin, and cirsilineol which act as potent antioxidant and antimicrobial agents that help to improve body weight gain (Kelm et al., 2000).

### Feed Conversion Ratio

We also observed an improved feed conversion ratio in the treatment groups where clove and tulsi were supplemented at 0.5 to 1% and 2 to 3%, respectively. This finding is similar to the previous reports (Cabuk et al., 2014; Al-Mufarrej et al., 2019; Arif et al., 2022; Naeem et al., 2022). Clove has potential antimicrobial and antioxidant properties (Batiha et al., 2020). Clove is rich in eugenol and different trace minerals which improve digestive functionality (Ghanima et al., 2020). Tulsi also exerts many beneficial therapeutic effects on the body through its antistress and antioxidant properties, antimicrobial and immunomodulatory actions, and gastroprotective effects (Eevuri and Putturu, 2013; Batiha et al., 2020). These effects of clove and tulsi might contribute to the improved performance of the broilers. However, a negative impact on growth performance was observed in the current study while clove and tulsi were supplied at a higher concentration. The level of feed intake may be a potential reason behind this. It may also be due to the changes in the intestinal epithelium, resulting from poor digestion and absorption of nutrients due to changes in the lining epithelium of the intestine. According to Kreydiyyeh et al. (2000), a high concentration of clove may inhibit the intestinal absorption of some nutrients.

### Carcass Characteristics

In the current study, we found higher carcass yield in the treatment groups supplemented with low to medium concentrations of clove and tulsi. This finding is in line with Vasanthakumar et al. (2013) and Cabuk et al. (2014) who reported high carcass yield in response to clove oil supplementation. Herbal products exert a beneficial impact on carcass yield which is attributed to the increased absorption of amino acids, utilization of dietary nutrients, and enhanced metabolism of proteins thus resulting in increased carcass yield (Mansoub, 2011). However, the decreased weight gain justifies the lower carcass weight in the high-dose group.

### Giblet and Offal Weight

The present study findings are supported by Bozkurt et al. (2012) who stated that the inclusion of 1 to 2% clove in the diet does not affect the weight of gizzard, liver, and abdominal fat depot. According to Cabuk et al. (2014) and Mustafa (2016), the weight of the gizzard, liver, and heart remains unaffected in birds fed clove with their diet. On the contrary, Gandomani et al. (2014) reported an increase in the relative weight of the liver, and spleen, and a decrease in case of the heart. However, we found an increased heart weight in the current study while the other organs remained almost unaffected by clove and tulsi supplements. This is in agreement with Hossain et al. (2021) who reported an increase in the relative weight of the broiler heart supplemented with neem and tulsi. However, Hossain et al. (2021) also reported significantly increased liver and spleen weight which contradicts our findings. In another study, Suliman et al. (2021) reported that a higher concentration (6%) of clove inclusion in broiler diets substantially increases the gizzard, liver, and heart weights in broilers.

### Gut Morphometry

The small intestine of a broiler is considered the major site for digestion and absorption of dietary nutrients (Svihus, 2014). The morphologic and morphometric properties of the intestine reflect the broilers' health status and are associated with the nutrient assimilation capacity as well as immunological functionality (Nicholson et al., 2012). In this study, we measured the gut morphometric indices to evaluate the functional changes that happened while fed different dose-dependent combinations of clove and tulsi. The lengths of different intestinal segments were significantly influenced by the treatments in the current study. This finding is similar to Al-Mufarrej et al. (2019) who reported an increased length of intestine while clove is supplemented at the rate of a 10 g/kg diet. However, clove supplementation reduces intestinal size while supplied at a concentration of 20 g/kg diet or more which also supports the current study findings (Al-Mufarrej et al., 2019). There is no report of such an increase in intestinal size in response to tulsi supplementation.

Any interpretation of changes in intestinal functionality based solely on intestinal size is not plausible. In this context, histomorphometric indices can be a more reliable indicator to assess intestinal health and function (Awad et al., 2011; Al-Baadani et al., 2016). In the current study, we observed augmented villus height which corresponds to the previous reports (Mohammadi et al., 2014; Mahdavi et al., 2014; Thuekeaw et al., 2022). Notwithstanding, Chowdhury et al. (2018) and Jahejo et al. (2019) reported that villus height remains unaffected while fed clove or tulsi. On the contrary, Al-Mufarrej et al. (2019) reported decreased villus height while the broilers were fed clove powder which contradicts our finding. However, the villi width and crypt depth were unaffected by the treatments which coincides with the earlier reports (Chowdhury et al., 2018; Jahejo et al., 2019). Villus surface area was also increased in the experimental groups treated with low to medium concentrations of clove and tulsi. Thuekeaw et al. (2022) also reported an increased villus surface area in broilers fed tulsi with their diet. Contrarily, Al-Mufarrej et al. (2019) demonstrated that the inclusion of clove powder with feed decreases the villus surface area. Increased villus surface area indicates an improvement in nutrient absorbability and gut health (Mohamed et al., 2014). According to the previous study reports, the intestinal digestibility and absorbability increase with the increase in absorptive surface area of the villi and thus augments the growth performance of broilers (Laudadio et al., 2012; Mohamed et al., 2014; Mahdavi et al., 2014). It is noteworthy that the mucosal thickness of the large intestine of the experimental broilers remained almost unaffected by the treatment which suggests that the beneficial effects of feeding clove and tulsi are largely centered on the small intestine of broilers. However, the gut morphometric indices coincide with the growth performance of the broilers.

### Microbial Status of the Cecum

Gut microbial population significantly affects the health status of the host. They benefit the host by facilitating nutrient exchange and modulating the digestive and immune systems (Pan and Yu, 2014; Clavijo and Flórez, 2018). Diarrhea is one of the most frequently occurring diseases in poultry resulting in high morbidity and mortality that is widely caused by *E. coli* (Wang et al., 2017; Liang et al., 2021). *Lactobacillus spp.* are the common inhabitants of the intestine and are widely used as probiotics for their roles in the competitive exclusion and inhibition of pathogenic bacteria like *E. coli* as well as in enhancing immunity (Wang et al., 2017; Xiang et al., 2022). So, the focus of the current study was centered on these two bacterial population in the broiler cecum. In this study, the *Lactobacillus* spp. population increased in the treated groups without affecting the total bacterial count which is in line with Agostini et al. (2012) and Chakma et al. (2020). On the contrary, the *E. coli* count decreased substantially in the treated groups. Eugenol is a major constituent of both clove and tulsi which has very potent antibacterial activity (Agostini et al., 2012; Eevuri and Putturu, 2013; Batiha et al., 2020). There are reports that phytogenic feed additives restrict the proliferation of pathogenic microorganisms in the gut (Wati et al., 2015; Chowdhury et al., 2018). Possibly the reduction of the *E. coli* population provided more room for the growth of beneficial microbes and thus facilitated *Lactobacillus* spp. proliferation (Wati et al., 2015). The increase of *Lactobacillus* spp. population and decreased *E. coli* count indicates improved gut health of the clove and tulsi treated broilers. These findings are also correlated with the growth performance of the treated broilers.

## CONCLUSION

The current study findings clearly indicate that the combined effects of clove and tulsi are dose-dependent. They have strong potential to augment the growth performance as well as the gut health of broilers if supplied in an optimum dose. In this study, a combination of 1% clove powder and 3% tulsi extract resulted in the improvement of feed intake, weight gain, feed efficiency, carcass weight, gut morphometric indices as well as gut health of broilers. Based on these findings, it is concluded that a combination of clove (1%) and tulsi (3%) can be used as a growth promoter as well as a potential substitute for AGPs. However, further study is recommended to investigate the biochemical and meat quality indices in broilers fed clove and tulsi.
